# Unconditional cash transfers to low-income preterm infants and their families: a pilot randomized controlled trial

**DOI:** 10.1038/s41372-025-02293-2

**Published:** 2025-04-11

**Authors:** Zoe M. Bouchelle, Timothy D. Nelin, Elizabeth G. Salazar, Sydney Ragland, Destiny Uwawuike, Joshua K. Radack, Andrea F. Duncan

**Affiliations:** 1 Department of Pediatrics and Center for Health Systems Research, Denver Health, Denver, CO, USA.; 2 PolicyLab, Children’s Hospital of Philadelphia, Philadelphia, PA, USA.; 3 Division of Neonatology, Department of Pediatrics, Children’s Hospital of Philadelphia, Philadelphia, PA, USA.; 4 Leonard Davis Institute of Health Economics, Philadelphia, PA, USA.; 5 University of Pennsylvania Perelman School of Medicine, Philadelphia, PA, USA.; 6 Morehouse School of Medicine, Atlanta, GA, USA.; 7 University of Pennsylvania, Philadelphia, PA, USA.; 8 These authors contributed equally: Zoe M. Bouchelle, Timothy D. Nelin.

## Abstract

**OBJECTIVE:**

Unconditional cash transfers (UCTs)—no strings attached monthly payments—to low-income families may reduce financial stress and improve health outcomes. We sought to determine the feasibility and acceptability of randomizing low-income caregivers of preterm infants to a high- or low-value UCT for 4 months.

**STUDY DESIGN:**

Parallel, pilot randomized controlled trial that was preregistered (ClinicalTrials.gov NCT05930327). We enrolled 24 birthing parent-infant dyads. The intervention was a $325 monthly UCT and the active control was a $25 monthly UCT.

**RESULT:**

The intervention was feasible and universally acceptable among families in the high-value cash transfer arm. Exploratory outcomes revealed a high degree of financial strain, stress, and depressive symptoms.

**CONCLUSION:**

This study provides feasibility, acceptability, and preliminary efficacy data to inform a future, larger trial to examine the impacts of UCTs to low-income birthing parents of preterm infants.

**CLINICAL TRIAL REGISTRATION:**

ClinicalTrials.gov ID NCT05930327.

## INTRODUCTION

Poverty is pervasive in America, with 1 in 6 children [1] growing up in households with incomes below the federal poverty line [2]. It is hypothesized that poverty contributes to poor health outcomes in children through two pathways—an “investment pathway”, through which poverty limits parental ability to invest time and resources into their child’s health and development, and a “stress pathway”, through which poverty causes parental and familial psychological stress that negatively impacts the child’s health and development (See supplemental Fig. 1) [3].

Preterm infants are one pediatric population for which exposure to poverty may be particularly detrimental, and thus poverty reduction may be especially beneficial. Low-income caregivers of preterm infants experience additional economic strain due to the out-of-pocket costs (such as transportation), lost wages, and employment insecurity associated with the initial neonatal intensive care unit (NICU) hospitalization [4] and subsequent healthcare needs of their infants after discharge [5]. Among preterm infants, low income is associated with worse long-term health outcomes and higher acute care utilization [6–9].

A growing body of literature suggests that monthly unconditional cash transfers (UCTs)—no strings attached monthly cash payments—to low-income families may be an effective intervention to reduce financial stress, improve psychological health, and improve children’s health outcomes [9–19]. However, current studies on cash transfers focus primarily on term infants with only a single pilot delivering direct financial assistance to low-income preterm infants [19–21].

To address this gap, we conducted a pilot randomized controlled trial to examine the feasibility and acceptability of randomizing low-income caregivers of preterm infants to either high-value ($325 per month) or low-value ($25 per month) UCTs for 4 months, hypothesizing that cash transfers would be both feasible and acceptable.

## METHODS

### Trial design and ethics approval

This was a parallel, pilot randomized controlled trial. The trial was approved by the University of Pennsylvania IRB and preregistered (ClinicalTrials.gov NCT05930327). We adhered to CONSORT guidelines for pilot RCTs (Supplement) [22].

### Eligibility criteria

Participants included birthing parent-infant dyads recruited from a level III neonatal intensive care unit (NICU) within 2 weeks of a preterm birth (< 37 weeks GA). Eligibility included caregiver Medicaid eligibility, age ≥18 years, a social security or tax identification number, fluency in English or Spanish, Philadelphia County residence, not planning adoption, and not likely to move out of state within a year. Possessing a social security or tax identification number was required for the distribution of the debit cards utilized in the study.

### Recruitment

Birthing parents were approached in person or over the phone within 2 weeks of the birth of their infant to participate in a study on their experiences caring for and managing the costs of having a preterm infant. They were informed that the study would consist of 1 baseline survey, 1 electronic survey at 2 months, 1 electronic survey at 4 months, and 1 semi-structured interview at 4 months, as well as abstraction of data from their and their child’s electronic health record (EHR). They were also informed that as part of the study, they would be offered monthly UCTs via a debit card for 4 months.

### Informed consent to participate

We employed a two-step consent process. The first consent served as consent to study procedures, with the disclosure that the study involved dispensation of cash payments, while the second consent occurred after randomization and was consent for receipt of the UCT. To address ethical concerns regarding the possibility that cash transfers might coerce birthing parents to participate in research-based data collection, informed consent to participate in the research was uncoupled from the agreement to receive the monthly cash transfer.

The informed consent for the UCTs included information on how to use the card, potential impacts on government benefits, and tax implications. All study participants received a webinar prepared by a community partner, the Philadelphia Office of Community Empowerment and Opportunity, which has experience providing benefits counseling through community-based cash transfer trials, to allow birthing parents to receive counseling and assistance regarding any potential impacts of receiving the cash transfers on their benefits or taxes. Birthing parents also received information to set up 1:1 benefits counseling sessions as desired. While the amount of cash received was not likely to alter benefits or taxes, in an abundance of caution, the research team also worked with the Department of Human Services to receive a letter for participants to submit to properly account for their cash transfers in conjunction with the Philadelphia Office of Community Empowerment and Opportunity. Eligible caregivers could then decide whether to enroll. Enrollment occurred from July through November 2023.

### Sample size

We determined a pragmatic sample size of 24 birthing parent-infant dyads to assess feasibility and acceptability.

### Intervention

The intervention was a $325 monthly UCT distributed via debit card over four months. The active control was a $25 monthly UCT distributed via debit card over four months. The first cash transfer was distributed at the time of the enrollment and the subsequent 3 were distributed within 2 days of the child’s monthly birthday.

The rationale for assigning the high-value cash group to $325 per month and the low-value cash group to $25 per month (a difference of $300 per month) is based on prior studies showing that $300 is a meaningful monthly amount for low-income children [23]. The amount is larger than the $250 per month child allowance recommended in the National Academy of Sciences 2019 report as the single most effective national strategy to reduce child poverty by 50% in 10 years [24]. It is similar to the monthly amount that is being delivered in the Baby’s First Year’s study (a difference of $313 per month) [20]. It is equal to the monthly amount that was delivered as part of the 2021 expanded Child Tax Credit ($300/month for children <5 years of age) which has been associated with meaningful differences in poverty and food insufficiency. Thus, we believed the $300 difference between the high-value and low-value cash group would be feasible, based on evidence-based recommendations, and may have a policy impact. Finally, we assigned a high-value and low-value cash group, instead of a cash group and control, recognizing that there may be bias introduced if only one group received a debit card and associated counseling.

### Randomization

Caregivers were randomized 1:1 to receive either high-value or low-value UCTs, using parallel block randomization stratified by gestational age (≥ 28 weeks or <28 weeks). The random allocation sequence was generated by a statistical analyst (JR) using R-statistical software. ZB aided in the random allocation sequence generation and was not blinded to the randomization schema. ZB created sequentially numbered envelopes based on the sequence. Authors ES, TN, SR, and DU enrolled participants and were blinded to the randomization schema by the envelopes. After assignment to interventions, no participants, care providers, or those assessing outcomes were blinded due to the nature of the intervention.

### Data collection

Baseline demographic information was collected via caregiver survey at the time of enrollment, including caregiver gender, race, ethnicity, marital status, cohabitation with one’s partner, number of adults and children in the home, highest educational level completed, and household gross income in the prior year. Though social constructs, we included race and ethnicity due to the contribution of systemic racism and discrimination to income inequality [25].

We also collected data on other covariates that lie along the hypothesized stress and investment pathways including the ability to manage a $400 unexpected expense, degree of worry about being able to meet monthly living expenses, difficulty paying utilities or electricity, lack of reliable transportation impacting the ability to carry out daily activities, costs impacting ability to access medical care, purchase of books or reading material for infant, working car ownership, lack of reliable transportation impacting NICU visitation, employment and parental leave, food insecurity, birthing parent self-reported overall health, birth parent report of infant’s overall health, birthing parent depression, birthing parent stress, and breastfeeding [24, 26, 27]. We also collected information on acceptability via the validated Acceptability of Intervention (AIM) measure [28]. While we initially intended to track visitation frequency to the NICU through a sign-in log, we did not collect these data after feedback from the study team, prospective participants, and the NICU staff that it may be unintentionally coercive.

Finally, we extracted data from the EHR on health outcomes and healthcare utilization, including infant birthweight, infant comorbidities, infant NICU length of stay, infant reliance on durable medical equipment at discharge, infant number of readmissions and ED visits within 3-months. While we did not preregister outcomes related to birthing parent healthcare utilization, we did consent birthing parents to extract EHR data on length of stay after delivery, post-partum care within 6 weeks of delivery, ED visits within 3 months of delivery, and inpatient readmissions within 3 months of delivery.

Though data on what families of preterm infants may spend money on may be helpful for providers and policy makers, we did not track what the cash was spent on to reduce bias that may occur with tracking spending. While we acknowledge that this information could have been collected upon completion, we prioritized UCT recipient autonomy given previous studies that demonstrated an influence in spending behavior based on monitoring, underscoring the importance of understanding the implementation context and potential biases introduced by monitoring [29, 30].

### Primary outcomes

Primary outcomes were feasibility and acceptability. Feasibility was defined as ≥60% enrollment and ≤30% attrition measured on 2- and 4-month surveys and payment feasibility was defined as ≥90% of monthly UCTs delivered within 2 days of the infant’s monthly birthdays.

Acceptability was defined as ≥90% “agreeing” or “strongly agreeing” to 3 measures on the validated Acceptability of Intervention (AIM) tool on the 2-month survey and ≥90% uptake of those eligible for UCTs to consent [28].

### Statistical analysis

Data are presented as means for continuous data and raw counts for categorical variables. As this is a feasibility study, no statistical tests were conducted. Analyses were completed using Stata 18 (StataCorp, College Station, Texas, USA).

## RESULTS

**Fig. 1:**
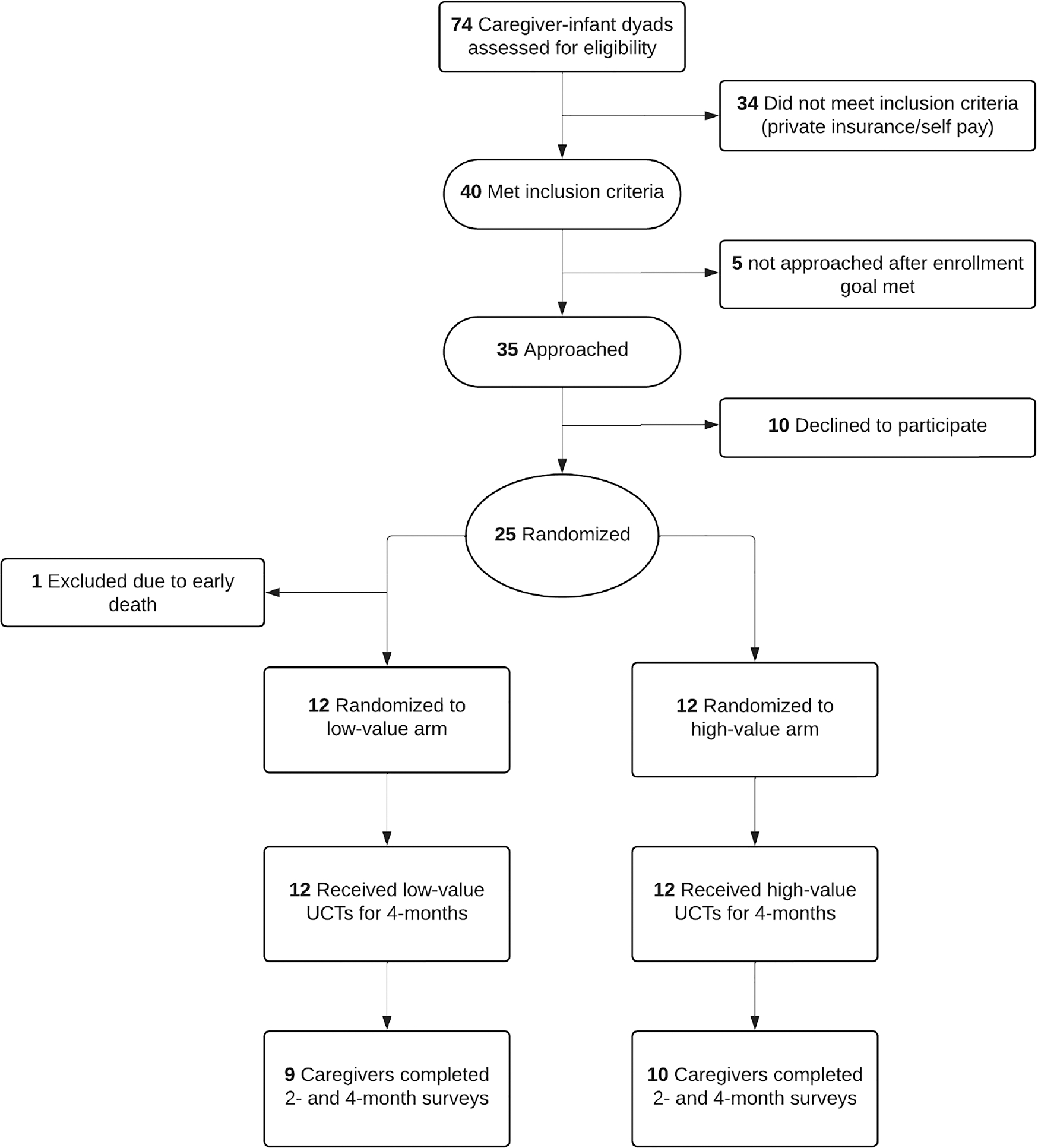
Consort diagram. Consort diagram for study recruitment and enrollment.

Twenty-five caregiver-infant dyads enrolled, with one excluded due to infant death (Fig. 1). Baseline demographics are displayed in Table 1. 20 birthing parents (83%) identified as Black or African American race and 3 (13%) as Hispanic. 16 birthing parents (67%) reported a household income less than $50,000. 12 birthing parents (50%) reported that they actively worked while they were pregnant, only 3 birthing parents (13%) took paid maternity leave after birth, and 15 (63%) owned a working car. Preterm infants in the study had a mean gestational age of 30 weeks and mean birthweight of 1562 g.

The intervention was feasible, with 25 of 35 (71%) approached caregivers enrolling and 19 (79%) caregivers completing the 2- and 4-month surveys (Fig. 1). We also established payment feasibility, with 93 of 96 (97%) payments made within 2 days of the infant’s monthly birthday.

For acceptability, 100% of caregivers in the high-value arm “agreed” or “strongly agreed” that UCTs met their approval, were appealing, and welcomed. In contrast, only 3 (33%) of caregivers in the low-value arm “agreed” or “strongly agreed” that UCTs met their approval, 5 (56%) “agreed” or “strongly agreed” that UCTs were appealing, and 5 (56%) “agreed” or “strongly agreed” that UCTs were welcomed.

Table 2 displays expanded survey data on covariates that lie along the hypothesized stress and investment pathways related to financial strain, caregiver physical and mental health, and caregiving. In addition, it includes data extracted from the EHR on infant health and healthcare utilization and birthing parent healthcare utilization.

Of note, across survey waves in each study arm, a minority of birthing parents report feeling “very confident” they could cover an unexpected $400 expense, a third or more worry about expenses “often” or “very often”, and a third or more report experiencing food insecurity in the last month. Across survey waves in each study arm, a minority of birthing parents had a passing score on the PHQ-2 depression screen or the Perceived Stress Scale, suggesting a high prevalence of symptoms of depression and stress. Despite financial stressors, a majority of caregiving reported purchasing books for their infant by the 4-month survey. Notably, 4 (40%) of birthing parents in the high-value UCT arm reported continuing to give their infant some breastmilk at 4 months, compared to none in the low-value arm. For infant health and healthcare utilization, 10 (77%) of infants in the high-value arm were up to date on their immunizations at 4 months, compared to 7 (58%) in the low-value arm. For birthing parent healthcare utilization, 2 (17%) of birthing parents in the high-value arm had an ED visit within 3 months of delivery, compared to 7 (58%) in the low-value arm.

## DISCUSSION

In this pilot randomized controlled trial in an urban level III NICU, we found that randomizing low-income birthing parents of preterm infants to either high-value ($325 per month) or low-value ($25 per month) UCTs for 4 months was a feasible intervention. We also found that acceptability of the intervention was universal among birthing parents in the high-value UCT arm, but lower among birthing parents in the low-value UCT arm. Finally, although our pilot was not powered to assess clinical outcomes, we found a high degree of financial strain, depressive symptoms, and stress symptoms among the birthing parents. This study provides feasibility, acceptability data and preliminary efficacy data to inform a future, larger trial powered to examine the impact of UCTs to low-income birthing parents of preterm infants on financial strain, psychological well-being, and infant health outcomes.

The difference in acceptability of the intervention between the high-value and low-value UCT arms is unexpected and has implications for future trials. Specifically, 100% of caregivers in the high-value arm “agreed” or “strongly agreed” that UCTs met their approval, were appealing, and welcomed. In contrast, only 3 (33%) of caregivers in the low-value arm “agreed” or “strongly agreed” that UCTs met their approval, 5 (56%) “agreed” or “strongly agreed” that UCTs were appealing, and 5 (56%) “agreed” or “strongly agreed” that UCTs were welcomed. One might expect that cash gifts of any amount would be highly acceptable; however, it appears that the $25 per month cash gifts were less so. This may be that the $25 per month cash gifts were viewed as too small to make a difference in the lives of families. It may also be that, knowing one could have been randomized to a higher-value arm, the $25 per month cash gifts became less acceptable. Future qualitative studies may explore this difference. In addition, future trials may enhance acceptability by employing a stepped wedge design to ensure all families receive the high-value intervention, albeit at different time points. Finally, future trials may consider not revealing to participants the monetary value of the alternative arm, though the ethical implications of this must be considered.

We found that the intervention was feasible, defined as ≥60% enrollment and ≤30% attrition measured on 2- and 4-month surveys and ≥90% of monthly UCTs delivered within 2 days of the infant’s monthly birthdays. Notably, although ≥90% of monthly UCTs were delivered within 2 days of the infant’s monthly birthdays, because payments were made manually by the study team, there was variation in the time of day and date on which the payment was made. In a larger trial, manual payments would not be feasible and instead would need to be automated. In addition, delivering payments at a set date and time would increase reliability for parents, who may be relying on the payments for certain expenses.

Although the trial was not powered to assess clinical outcomes, we did note four findings among our exploratory outcomes. First, there was a high degree of financial strain among the families, with a minority of birthing parents reporting feeling “very confident” they could cover an unexpected $400 expense, approximately a third or more worrying about expenses “often” or “very often”, and a third or more reporting experiencing food insecurity in the last month. This finding aligns with a prior study, which found that among a diverse sample of US households with a high prevalence of poverty, ≥60% of families with preterm children younger than 24 months experienced 1 or more unmet basic needs [31]. It also aligns with a 2023 study using data from the National Survey of Children’s Health, finding that material hardships were exceptionally common among US preterm children, where 41–48% of preterm VLBW children and 20–28% of preterm LBW children lived in households with food insufficiency, financial hardships, or difficulty paying medical bills [32]. Collectively, these findings underscore the high prevalence of material hardship among households with preterm infants and the need for policies and programs to address these hardships to promote health and health equity.

Second, we found that a minority of birthing parents had a passing score on the Perceived Stress Scale, suggesting a high prevalence of symptoms of stress. On the PHQ-2, 11 (92%) of parents in the high-value UCT group had a passing score on the baseline survey, however on remaining survey waves in both the high and low UCT groups, between 44–67% of parents had a passing score, suggesting a high prevalence of symptoms of depression. While our pilot RCT was not powered to detect differences in exploratory outcomes and the decrease in passing score over time was unexpected, we hypothesize that NICU admission may increase caregiver stress and increase the likelihood of depressive symptoms. However, other data to suggest that cash transfers, even over a short duration, may have positive impacts on parental mental health. For example, Kovski et al. report reduced anxiety and depression symptoms among parents with low incomes in the United States after the expansion of the 2021 Child Tax Credit [33]. More research is needed to examine the link between cash transfers and mental health among households with preterm infants.

Third, we found that 4 (40%) of birthing parents in the high-value UCT arm reported continuing to give their infant some breastmilk at 4 months, compared to none in the low-value arm. Breastmilk is associated with numerous potential benefits in preterm infants, including reduced incidence of necrotizing enterocolitis, reduced risk of immune-mediated illness, and improved neurodevelopmental outcomes [34]. These findings align with a prior pilot RCT suggesting weekly financial support increased skin-to-skin care and breastfeeding in a Massachusetts NICU [35]. We hypothesize that high-value UCTs may act through the “investment pathway” to enable birthing parents to allot specific time to breastfeeding. The potential of unconditional cash transfers to boost breastfeeding deserves more study.

Fourth and finally, we found that 2 (17%) of birthing parents in the high-value UCT arm had an ED visit within 3 months of delivery, compared to 7 (58%) in the low-value arm. While this pilot is not powered to detect differences in exploratory outcomes, there are plausible mechanisms through which cash transfers may decrease acute care utilization. Cash transfers may reduce financial stress, allowing birthing parents to access needed preventative care. Cash transfers may also reduce psychological stress, which is linked to physical health. More work is needed to examine the link between cash transfers and acute care utilization for both preterm infants and their caregivers.

There are several potential and feasible policy options exist to implement cash transfers in the U.S. for low-income families with preterm infants. First, Medicaid could deliver cash transfers that target the preterm population if cash transfers were permitted through Section 1115 demonstration waivers, which allow for innovation in the Medicaid program. Second, Federal Supplemental Security Income payments could be modified to expand eligibility to more families of preterm infants, to allow payments to occur sooner and not cap payments at $30 per month during the initial NICU stay, when families may be experiencing a high degree of financial stress [23]. Third, there is an opportunity for increased public-private partnerships to provide cash transfers, in a conditional or unconditional form, as an intervention to improve health outcomes and promote family health.

The trial has many strengths. First, it is a pilot of a novel intervention in this patient population, preceded by only a single pilot to our knowledge [19]. Second, the amount or “dose” of cash in the high-value and low-value arms was evidence-based and policy-relevant. Lastly, the study design included a two-step consent process, intervention and active comparator arms, and did not track how money was spent by caregivers, which may reduce bias.

This pilot also has limitations. First, this was a single-center pilot RCT in Philadelphia, which may affect generalizability. Second, the UCTs were intentionally designed to occur for 4 months, which for many families included time in the NICU and after discharge. This design, applied to a larger trial, would limit the interpretation of how UCTs may impact health outcomes when delivered at different time points. Third, participants required a social security or tax identification number for distribution of the debit cards which may be limiting if applied to a larger trial. Importantly, no birthing parents were excluded from this pilot due to lacking a social security or tax identification number. Fourth, we designed all study materials and surveys to be inclusive of participants who speak English or Spanish. Despite this, we were not able to enroll any families who spoke Spanish exclusively during this enrollment period and thus were not able to test the Spanish-language materials in our pilot. In a larger trial, we aim to expand eligibility to families speaking additional languages other than English to promote equity and inclusion.

## CONCLUSION

In this pilot randomized controlled trial, we found that randomizing low-income birthing parents of preterm infants to either high-value or low-value unconditional cash transfers for 4 months was a feasible intervention that was universally acceptable among birthing parents in the high-value UCT arm. Although our pilot was not powered to assess clinical outcomes, we found a high degree of financial strain, depressive symptoms, and stress symptoms among the birthing parents. This study provides new feasibility and acceptability data and preliminary efficacy data to inform a future, larger trial powered to examine the impact of unconditional cash transfers to low-income birthing parents of preterm infants on financial strain, psychological well-being, and infant health outcomes.

## Supplementary Material

Supplement

**Supplementary information** The online version contains supplementary material available at https://doi.org/10.1038/s41372-025-02293-2.

## Figures and Tables

**Table 1.** Baseline birthing parent and infant characteristics.

Birthing parent characteristics	Total (n = 24)	Low-Value UCT (n = 12)	High-value UCT (n = 12)
	n (%)	n (%)	n (%)
Race			
American Indian or Alaska Native	1 (4)	1 (8)	0 (0)
Black or African American	20 (83)	10 (83)	10 (83)
Multiple Races	1 (4)	0 (0)	1 (8)
White	1 (4)	0 (0)	1 (8)
Prefer not to answer	1 (4)	1 (8)	0 (0)
Hispanic Ethnicity	3 (13)	2 (16)	1 (8)
Household size, mean (SD)	3.8 (1.6)	3.7 (1.4)	3.9 (1.8)
Live with spouse or partner	11 (46)	5 (42)	6(50)
Education			
Less than high school	3 (13)	1 (8)	2 (16)
High school diploma or GED	11 (46)	6 (50)	5 (42)
Trade or technical school	2 (8)	2 (8)	0 (0)
Associate's degree	6(25)	2 (8)	4(33)
Bachelor's degree	2 (8)	1 (8)	1 (8)
Household income			
$49,999 or less	16 (67)	7 (58)	9 (75)
$50,000 to $99,999	5 (21)	4 (33)	1 (8)
Prefer not to answer	3 (13)	1 (8)	2 (16)
Own a car in the household	15 (63)	8 (67)	7 (58)
Actively worked while pregnant	12 (50)	7 (58)	5 (42)
Took paid maternity leave after birth	3 (13)	1 (8)	2 (16)
Difficulty paying utility bill in the last month	10 (42)	5 (42)	5 (42)
Lack of reliable transportation in the last month	6 (25)	3 (25)	3 (25)
*Infant Characteristics*	Total (n = 25)	Low-value UCT (n = 12)	High-value UCT (n = 13)^a^
Gestational age, weeks, mean (SD)	30 (4)	30 (4)	30 (4)
Birthweight, g, mean (SD)	1562 (772)	1514 (737)	1603 (829)

*SD* standard deviation.

a 13 infants in high-value group as 1 birthing parent gave birth to twins.

**Table 2.** Exploratory outcomes.

*Survey Data*	Low-value cash transfer n (%)			High-value cash transfer n (%)		
*Survey Wave.*	*Baseline*	*2-month*	*4-month*	*Baseline*	*2-month*	*4-month*
*Financial Strain*	(n = 12)	(n = 9)	(n = 9)	(n = 12)	(n = 10)	(n = 10)
“Very confident" can cover a $400 expense	4(33)	0 (0)	0 (0)	2 (17)	2 (20)	1 (11)
Worry about expenses “often” or “very often”	5 (42)	3 (33)	3 (33)	5 (42)	5 (50)	3 (30)
Food insecurity in the last month	5 (42)	4 (44)	3 (33)	2 (17)	6 (60)	3 (30)
**Caregiver Mental and Physical Health**						
Birthing parent PHQ-2 passing score (<3)	8 (67)	4 (44)	5 (56)	11 (92)	5 (50)	5 (50)
Birthing parent Perceived Stress Scale passing score (< 6)	5 (42)	2 (22)	1 (11)	4(33)	2 (20)	4 (40)
Healthcare costs impacting ability to access medical care ^a^	n/a	0 (0)	0 (0)	n/a	1 (10)	0 (0)
Rate overall health as “excellent”, “very good”, or “good” ^b^	6(50)	n/a	5 (56)	7 (58)	n/a	6 (60)
**Infant Caregiving**						
Lack of transportation impacted ability to visit NICU ^c^	1 (8)	2 (22)	n/a	2 (17)	2 (20)	n/a
Purchased books for newborn infant ^a^	n/a	4 (44)	6 (66)	n/a	6 (60)	7 (70)
Rate infant health as “excellent”, “very good”, or “good” ^d^	n/a	7 (78)	n/a	n/a	7 (70)	n/a
Receipt of any breastmilk ^e^	n/a	n/a	6 (67)	n/a	n/a	8 (80)
Continued receipt of breastmilk at 4 months ^e^	n/a	n/a	0 (0)	n/a	n/a	4 (40)
Electronic Health Record Data	Low-Value Cash Transfer n (%)			High-Value Cash Transfer n (%)		
*Infant Health and Healthcare Utilization*	(n = 12)			(n = 13) ^f^		
Up-to-date with 4-month immunizations	7 (58)			10 (77)		
Treatment for retinopathy of prematurity	1 (8)			0 (0)		
Necrotizing enterocolitis	0 (0)			1 (8)		
Grade 2 or 3 bronchopulmonary dysplasia	3 (25)			5 (38)		
Grade III or IV intraventricular hemorrhage	2 (17)			0 (0)		
Birthing hospital length of stay, days, mean (SD)	39 (36)			61 (56)		
Home Oxygen, Tracheostomy, or Gastrostomy Tube	0 (0)			1 (8)		
Readmission within 3-months of NICU discharge	1 (8)			1 (8)		
ED Visits within 3-months of NICU discharge	5 (42)			4(31)		
*Birthing Parent Healthcare Utilization*	(n = 12)			(n = 12)		
Length of stay, days, mean (SD)	8 (4)			5 (2)		
Post-partum care within 6-weeks of delivery	7 (58)			8 (67)		
ED visits within 3-months of delivery	7 (58)			2 (17)		
Inpatient readmissions within 3-months of delivery	3 (25)			1 (8)		

*SD* standard deviation, *PHQ-2* patient health questionnaire-2.

a Asked on 2- and 4-month survey instrument.

b Asked on baseline and 4-month survey instrument.

c Asked on baseline and 2-month survey instrument.

d Asked exclusively on 2-month survey instrument.

e Asked exclusively on 4-month survey instrument.

f 13 infants in high-value group as 1 birthing parent gave birth to twins.

## Data Availability

The de-identified data that support the findings of this study are available from the corresponding author upon reasonable request. Data are located in controlled access data storage at the Children’s Hospital of Philadelphia.
