# Supplementary material for: Unconditional cash transfers to low-income preterm infants and their families: a pilot randomized controlled trial
Source: J Perinatol. 2025 Apr 11;45(9):1233–9. doi: 10.1038/s41372-025-02293-2 (PMC12326556; doi:10.1038/s41372-025-02293-2)
Supplement: Supplementary file 1 — Supplement [file 41372_2025_2293_MOESM1_ESM.pdf]

Supplemental Online Content

I. **Conceptual Model**
II. **Intervention, Subject Withdrawal, Minimizing Risks**
III. **Survey Measures**
IV. **Statistical Methods and Pre-analysis Plan**
V. **CONSORT Checklist**
VI. **References**

This supplemental material has been provided by the authors to give readers additional information about their work.

I. Conceptual Model

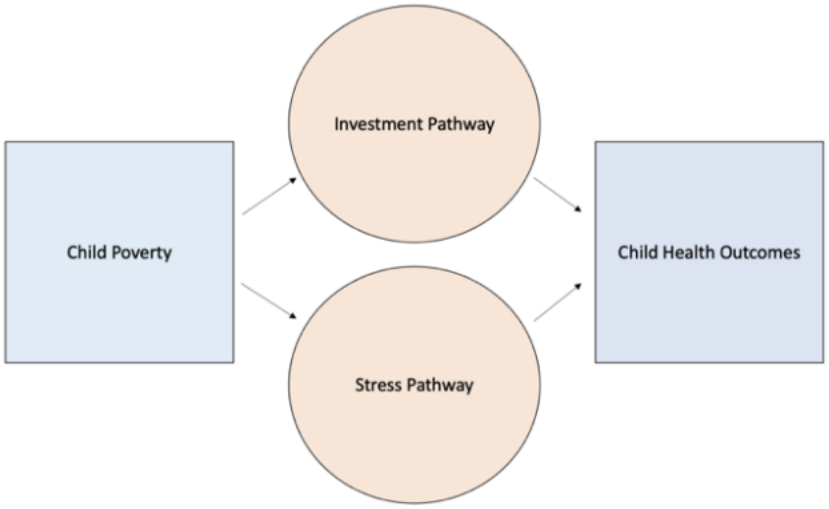

II. Intervention, Subject Withdrawal, Minimizing Risks

Intervention

We randomized 12 birthing parents to the treatment arm (receiving $325/month for 4 months via debit card) and 12 birthing parents to the active comparator arm (receiving $25/month for 4 months via debit card) for a total of 24 infant-caregiver dyads. We utilized Greenphire (greenphire.com/home) to administer the payments and provide toll-free customer service. Their pre-paid debit card does not require a bank account and can be used to make purchases directly at stores ("point-of-service") or to withdraw cash at ATMs or banks. All participants received information on the logistics of using the debit card and the procedure for checking their balances, as well as reporting lost or stolen cards. The monthly cash transfers were disbursed to the Greenphire debit card each month, on or around their child's date of birth, for 4 months.

Birthing parents received the 4 months of cash transfers regardless of whether they are lost to follow-up and will still be able to use the debit card after the study is completed.

Subject Withdrawal

Subjects could withdraw from the study or cash transfers at any time without prejudice to their care. Subjects could also be discontinued from the study or cash transfers at the discretion of the investigators due to abusive or disrespectful treatment of study staff (which did not occur).

Cash payments and data collection were not stopped in the event of a transfer to another facility. Cash payments were stopped in the event of a neonatal death as the ClinCards were distributed in the name of the infant and we did not want to cause additional trauma by continuing to deliver cash transfers in the name of the infant.

Cash payments did not stop in the event of a birthing parent's death (which did not occur).

Minimizing Risks

<u>Parental discomfort with questions:</u> The birthing parents were made aware of the types of questions that will be asked during the informed consent process and reminded that participation is voluntary. Birthing parents were given the phone number to call a study team member for concerns. We informed birthing parents that there were no right or wrong answers and that they could decline to answer any questions that they did not feel comfortable answering.

<u>Participants' time:</u> We explained the time requirements for each survey and provided each birthing parent with $25 compensation each for the baseline, 2-month, and 4-month surveys each and $40 if they participated in the qualitative interviews.

We were sensitive to the physical and emotional distress that caregivers may be experiencing when we approached them for participation. Specifically, we checked in with their nurses before approaching parents to gauge when would be a good time to approach the family, if at all. We also included language when approaching the family that recognized the difficulty of the time and ensured families that speaking with the research team was optional and if they did not wish to speak to the team or would prefer the team return at another time, then we would do everything we could to accommodate their schedule and preferences. Finally, we offered the families a welcome bag with a bottle of water and small snack to ensure that they had access to something to drink and eat to make them as comfortable as possible.

<u>Eliciting depression</u>: We administered the validated PHQ-2 at baseline, 2-months, and 4-months.
If the birthing parent had a score of 3 or more on the PHQ-2, this triggered a prompt with information on resources through Community Behavioral Health and a crisis hotline.

<u>Impact of cash on eligibility for other programs</u>: In the consent form for the cash intervention, we informed families about the potential effect of the cash transfers on their program eligibility. We also provided contact information for a local partner, The Philadelphia Office of Community Empowerment and Opportunity, which has experience providing benefits counseling through community-based UCT trials. The research team also worked with the Department of Human Services to receive a letter for participants to submit to properly account for their cash gifts. No participants reported loss of government benefits to our study team.

III. Survey Measures

Baseline Survey

First, we will collect some contact information:

Name
Phone number 1
Phone number 2
Phone number 3
Email Address 1
Email Address 2

Next, we will ask some questions about you and your family.

What is your date of birth?

What gender do you identify as? (Multiple choice)
• Male
• Female
• Other (Non-binary, agender, gender-fluid, etc):

What race do you identify as? (Multiple choice, select all the apply)
• White
• Black or African American
• American Indian or Alaska Native or First Nations
• Asian
• Native Hawaiian and Other Pacific Islander
• Two or more races
• Some other race:

What ethnicity do you identify as? (Multiple choice)
• Hispanic, Latino, or Spanish origin
• Not of Hispanic, Latino, or Spanish origin

Please list the ages of the children that live with you.

Not including yourself, how many other adults (people 18 or older) live with you?

What is your marital status? (Multiple choice)
• Never married
• Single living together
• Married
• Separated
• Divorced
• Widowed
• Other

Do you live with your spouse or partner?
• Yes
• No

What is the highest level of education you have completed? (Multiple choice)
• Less than High School Education
• High school diploma or GED (diploma equivalency test)
• Trade or technical school
• Associate's Degree (2-year college degree)
• Bachelor's Degree (4-year college degree)
• Advanced or Professional Degree (Masters, PhD, MD)

Do you own a car that works?
• Yes
• No

Please indicate how much you agree with the following statement. Since my baby was born, lack of reliable transportation has kept me from visiting my baby in the NICU.
• Strongly Agree
• Agree
• Neither Agree nor Disagree
• Disagree
• Strongly Disagree

Next, we will ask some questions about your households' finances and benefits you may receive.

What was your household's total income before taxes and deductions for 2021? Please include income from all wage earners and do not include what you may receive in government benefits.

Did you work for pay at any time while you were pregnant with your baby?
Source: Baby's First Years
Yes
No

Did you continue working until your baby was born or did you stop working earlier than that?
Source: Baby's First Years
Continued working until birth
Stopped working before birth

Once you stopped working, before your baby was born, were you still being paid?
Source: Baby's First Years
Yes – continued to get paid after stopped working
No – once I stopped working, I stopped getting paid

Do you plan to take maternity leave, either paid or unpaid, now that your baby has been born?
Source: Baby's First Years
Yes – Paid Maternity leave
Yes – Unpaid Maternity leave
Maternity leave is not available to me
No

Please indicate which services and government benefits you currently receive (select all that apply):
Source: Baby's First Years
• Food stamps or Supplemental Nutrition Assistance Program (SNAP / EBT)
• Free or reduced childcare
• Early Head Start
• Head Start
• Women, Infants and Children (WIC)
• State Unemployment
• Cash assistance/ Temporary Assistance for Needy Families (TANF)
• Medicaid coverage for self
• Housing assistance
• Heating /Air Conditioning assistance or Low-Income Home Energy Assistance Program (LIHEAP)
• Social Security Disability Insurance (SSDI)
• Other

How confident are you that you could come up with $400 if an unexpected expense arose within the next month?
Source: Well-being and Needs Survey
• Not at all confident
• Not too confident
• Somewhat confident
• Very confident

How often do you worry about being able to meet your monthly living expenses?
Source: Baby's First Years
• All the time

• Very frequently
• Occasionally
• Rarely
• Very rarely
• Never

Next, we want to ask you about your health.
Aside from your stay at the hospital for the birth of your baby, overall, how would you describe your health?
Source: Baby's First Years
• Excellent
• Very good
• Good
• Fair
• Poor

Next, we would like to ask some questions about getting food for your family.
Source: Adapted from Household Food Insecurity Access Scale (HFIAS) for Measurement of Food Access

In the past month, did you worry that your household would not have enough food?
• Yes
• No

In the past month, were you or any household member not able to eat the kinds of foods you preferred because of a lack of resources?
• Yes
• No

In the past month, did you or any household member have to eat some foods that you really did not want to eat because of a lack of resources to obtain other types of food?
• Yes
• No

In the past month, did you or any other household member have to eat less in a day because there was not enough food?
• Yes
• No

Next, we would like to ask some questions about various other costs families face.
In the past month, have you had trouble paying your heating bill and/or electricity bill?
• Yes
• No

In the past month, has lack of reliable transportation kept you from getting things needed for daily living?
• Yes
• No

Next, we would like to ask some questions about your feelings and thoughts.
Over the last *2 weeks*, how often have you been bothered by any of the following problems?
Source: PHQ-2
Little interest or pleasure in doing things
• Not at all
• Several Days
• More than Half of the Days
• Nearly Every Day
• Strongly Disagree

Feeling down, depressed, or hopeless
• Not at all
• Several Days
• More than Half of the Days
• Nearly Every Day
• Strongly Disagree

We would now like to focus on your feelings and thoughts in the past month. In each case, you will be asked to indicate by selecting how often you felt or thought a certain way.
In the last month, how often have you felt that you were unable to control the important things in your life?
Source: Perceived Stress Scale
• Never
• Almost Never
• Sometimes
• Fairly Often
• Very Often

In the last month, how often have you felt confident about your ability to handle your personal problems?
Source: Perceived Stress Scale
• Never
• Almost Never
• Sometimes
• Fairly Often
• Very Often

In the last month, how often have you felt that things were going your way?
Source: Perceived Stress Scale
• Never
• Almost Never
• Sometimes
• Fairly Often
• Very Often

In the last month, how often have you felt difficulties were piling up so high that you could not overcome them?
Source: Perceived Stress Scale
• Never
• Almost Never
• Sometimes
• Fairly Often
• Very Often

2-Month Survey
First, we will confirm your contact information:

Name
Phone number 1
Phone number 2
Phone number 3
Email Address 1
Email Address 2

Next, we will ask some questions about your households' finances and benefits you may receive.

How confident are you that you could come up with $400 if an unexpected expense arose within the next month?
Source: Well-being and Needs Survey
• Not at all confident
• Not too confident
• Somewhat confident
• Very confident

How often do you worry about being able to meet your monthly living expenses?
Source: Baby's First Years
• All the time
• Very frequently
• Occasionally
• Rarely

• Very rarely
• Never

Next, we want to ask you about your health.
Overall, how would you describe your health?
Source: Baby's First Years
• Excellent
• Very good
• Good
• Fair
• Poor

Next, we would like to ask some questions about getting food for your family.
Source: Adapted from Household Food Insecurity Access Scale (HFIAS) for Measurement of Food Access
In the past month, did you worry that your household would not have enough food?
• Yes
• No

In the past month, were you or any household member not able to eat the kinds of foods you preferred because of a lack of resources?
• Yes
• No

In the past month, did you or any household member have to eat some foods that you really did not want to eat because of a lack of resources to obtain other types of food?
• Yes
• No

In the past month, did you or any other household member have to eat less in a day because there was not enough food?
• Yes
• No

Next, we would like to ask some questions about various other costs families face.

In the past month, have you had trouble paying your heating bill and/or electricity bill?
• Yes
• No

In the past month, has lack of reliable transportation kept you from getting things needed for daily living?
• Yes
• No

Please indicate how much you agree with the following statement. In the past month, lack of reliable transportation has kept me from visiting my baby in the NICU.
• Strongly Agree
• Agree
• Neither Agree nor Disagree
• Disagree
• Strongly Disagree

Next, we would like to ask some questions about your feelings and thoughts.

Over the last *2 weeks*, how often have you been bothered by any of the following problems?
Source: PHQ-2
Little interest or pleasure in doing things
• Not at all
• Several Days
• More than Half of the Days
• Nearly Every Day
• Strongly Disagree

Feeling down, depressed, or hopeless
• Not at all
• Several Days
• More than Half of the Days
• Nearly Every Day
• Strongly Disagree

We would now like to focus on your feelings and thoughts in the past month. In each case, you will be asked to indicate by selecting how often you felt or thought a certain way.

In the last month, how often have you felt that you were unable to control the important things in your life?
Source: Perceived Stress Scale
• Never
• Almost Never
• Sometimes
• Fairly Often
• Very Often

In the last month, how often have you felt confident about your ability to handle your personal problems?
Source: Perceived Stress Scale
• Never
• Almost Never
• Sometimes
• Fairly Often
• Very Often

In the last month, how often have you felt that things were going your way?
Source: Perceived Stress Scale
• Never
• Almost Never
• Sometimes
• Fairly Often
• Very Often

In the last month, how often have you felt difficulties were piling up so high that you could not overcome them?
Source: Perceived Stress Scale
• Never
• Almost Never
• Sometimes
• Fairly Often
• Very Often

4-Month Survey
First, we will confirm your contact information:

Name
Phone number 1
Phone number 2
Phone number 3
Email Address 1
Email Address 2

Next, we will ask some questions about your households' finances and benefits you may receive.

Please indicate which services and government benefits you currently receive (select all that apply):
Source: Baby's First Years
• Food stamps or Supplemental Nutrition Assistance Program (SNAP / EBT)
• Free or reduced childcare
• Early Head Start
• Head Start
• Women, Infants and Children (WIC)

• State Unemployment
• Cash assistance/ Temporary Assistance for Needy Families (TANF)
• Medicaid coverage for self
• Housing assistance
• Heating /Air Conditioning assistance or Low-Income Home Energy Assistance Program (LIHEAP)
• Social Security Disability Insurance (SSDI)
• Other

How confident are you that you could come up with $400 if an unexpected expense arose within the next month?
Source: Well-being and Needs Survey
• Not at all confident
• Not too confident
• Somewhat confident
• Very confident

How often do you worry about being able to meet your monthly living expenses?
Source: Baby's First Years
• All the time
• Very frequently
• Occasionally
• Rarely
• Very rarely
• Never

Next, we want to ask you about your health.
Overall, how would you describe your health?
Source: Baby's First Years
• Excellent
• Very good
• Good
• Fair
• Poor

Next, we would like to ask some questions about getting food for your family.
Source: Adapted from Household Food Insecurity Access Scale (HFIAS) for Measurement of Food Access
In the past month, did you worry that your household would not have enough food?
• Yes
• No

In the past month, were you or any household member not able to eat the kinds of foods you preferred because of a lack of resources?
• Yes
• No

In the past month, did you or any household member have to eat some foods that you really did not want to eat because of a lack of resources to obtain other types of food?
• Yes
• No

In the past month, did you or any other household member have to eat less in a day because there was not enough food?
• Yes
• No

Next, we would like to ask some questions about various other costs families face.
In the past month, have you had trouble paying your heating bill and/or electricity bill?
• Yes
• No

In the past month, has lack of reliable transportation kept you from getting things needed for daily living?
• Yes

• No

Please indicate how much you agree with the following statement. In the past month, lack of reliable transportation has kept me from visiting my baby in the NICU.
• Strongly Agree
• Agree
• Neither Agree nor Disagree
• Disagree
• Strongly Disagree

Next, we would like to ask some questions about your feelings and thoughts.

Over the last *2 weeks*, how often have you been bothered by any of the following problems?
Source: PHQ-2
Little interest or pleasure in doing things
• Not at all
• Several Days
• More than Half of the Days
• Nearly Every Day
• Strongly Disagree

Feeling down, depressed, or hopeless
• Not at all
• Several Days
• More than Half of the Days
• Nearly Every Day
• Strongly Disagree

We would now like to focus on your feelings and thoughts in the past month. In each case, you will be asked to indicate by selecting how often you felt or thought a certain way.

In the last month, how often have you felt that you were unable to control the important things in your life?
Source: Perceived Stress Scale
• Never
• Almost Never
• Sometimes
• Fairly Often
• Very Often

In the last month, how often have you felt confident about your ability to handle your personal problems?
Source: Perceived Stress Scale
• Never
• Almost Never
• Sometimes
• Fairly Often
• Very Often

In the last month, how often have you felt that things were going your way?
Source: Perceived Stress Scale
• Never
• Almost Never
• Sometimes
• Fairly Often
• Very Often

In the last month, how often have you felt difficulties were piling up so high that you could not overcome them?
Source: Perceived Stress Scale
• Never
• Almost Never
• Sometimes
• Fairly Often

• Very Often

Next, we would like to ask some questions about your baby.
Overall, how would you describe your baby's health?
• Excellent
• Very good
• Good
• Fair
• Poor

There are many reasons people do not get medical care. During the past 4 months, was there any time when you or your baby needed medical care but did not get it because of costs?
• Yes
• No

Did you ever breastfeed or give your baby breastmilk?
Source: Baby's First Years
• Yes
• No

Are you currently breastfeeding or giving your baby breastmilk?
• Yes
• No

How many months did you breastfeed or give your baby breastmilk?
Source: Baby's First Years

Next, we would like to ask some questions about recent purchases.
In the last month, have you or any member of your household purchased: Any books or reading material for your baby?
• Yes
• No

Finally, we would like to learn more about your thoughts regarding this study.
Receiving the monthly cash payments during the first months of my baby's life met my approval.
Source: Acceptability of Intervention Measure
1 Completely Disagree
2 Disagree
3 Neither agree nor disagree
4 Agree
5 Completely Agree

Receiving the monthly cash payments during the first months of my baby's life was appealing to me.
Source: Acceptability of Intervention Measure
1 Completely Disagree
2 Disagree
3 Neither agree nor disagree
4 Agree
5 Completely Agree

I liked receiving the monthly cash payments during the first months of my baby's life.
Source: Acceptability of Intervention Measure
1 Completely Disagree
2 Disagree
3 Neither agree nor disagree
4 Agree
5 Completely Agree

I welcomed receiving the monthly cash payments during the first months of my baby's life.
Source: Acceptability of Intervention Measure
1 Completely Disagree
2 Disagree

3 Neither agree nor disagree
4 Agree
5 Completely Agree

IV. Statistical methods and Pre-Analysis Plan

Our primary outcomes were feasibility and acceptability. We defined feasibility as:

- ≥60% of those eligible enrolling in the study procedures
- ≥90% of those eligible for cash transfers consent to cash transfers
- ≥90% of monthly payments being delivered within 2 days on either side of the infant's monthly birthdays
- ≤30% attrition for the 2-month survey
- ≤30% attrition for the 4-month survey

We defined acceptability as ≥90% of respondents reporting "agree" to "strongly agree" to each of the 4 measures of acceptability.

We also defined exploratory outcomes. These include:

Birthing parent psychological distress

- PHQ-2 at baseline, 2-months, and 4-months
  - 2 item questionnaire using Likert Scale, scored 0-3, additive score for 2 questions, with the lowest score being 0 and the highest score being 6
  - Measure difference in the average between two groups at baseline , 2-months, and 4-months
- Perceived stress scale at baseline and, 2-months, and 4-months
  - 4 item questionnaire using Likert Scale, scored 0-5, with the lowest additive score being 0 and the highest additive score being 16
  - Measure the difference in the average between the two groups at baseline , 2-months, and 4-months

Birthing parent financial stress

- Ability to manage a $400 unexpected expense at baseline, 2-months, and 4-months
  - Single item using a Likert Scale, scored 0-3
  - Measure the difference in the average between the two groups at baseline, 2-months, and 4-months
- Degree of birthing parent worry about being able to meet monthly living expenses at baseline, 2-months, and 4-months
  - Single item using a Likert Scale, scored 0-5
  - Measure the difference in the average between the two groups at baseline, 2-months, and 4-months
- Visitation frequency to the NICU*
  - We intended to record visitation to the NICU using a visitation log, measuring visits/week beginning after first cash transfer disbursement through NICU discharge or 4 months after birth, whichever comes sooner and plot the median and average visits/week over time, overall and stratified by treatment arm and gestational age bracket.
  - *We did not track visitation frequency to the NICU after feedback from the study team, prospective participants, and the NICU staff as to not induce a feeling of coercion.

We also collected data on other outcomes that lie along the hypothesized stress and investment pathways to test and refine our data collection methods and instruments:

- Difficulty paying utilities or electricity at baseline
  - Single item, using a Likert Scale, scored 0-4
  - Measure the difference in the average between the two groups at baseline
- Lack of reliable transportation impacting ability to carry out daily activities at baseline
  - Single item, using a Likert Scale, scored 0-4
  - Measure the difference in the average between the two groups at baseline
- Costs impacting ability to access medical care at baseline, 2-months, and 4-months
  - Single item, using a Likert Scale, scored 0-4
  - Measure the difference in the average between the two groups at baseline, 2-months, and 4-months
- Purchase of books or reading material for infant at 2-months and 4-months
  - Single item, yes or no
  - Measure the difference in caregivers reporting 'yes' at 2-months and 4-months
- Working car ownership at baseline

    - Single item, yes or no
    - Measure the difference in caregivers reporting ‘yes’ at baseline
- Lack of reliable transportation impacting NICU visitation at baseline
    - Single item, using a Likert Scale, scored 0-4
    - Measure the difference in the average between the two groups at baseline
- Employment and Parental Leave at baseline at baseline
    - 4 item questionnaires, yes or no
    - Measure the difference in caregivers reporting ‘yes’ between the two groups at baseline
- Food insecurity at baseline, 2-months, and 4-months
    - 4 item questionnaires, yes or no
    - Measure the difference in caregivers reporting ‘yes’ between the two groups at baseline, 2-months, and 4-months

We also collected data on other health outcomes and healthcare utilization:

- Birthing parent report of infant’s overall health at 2- months
    - Single item, using a Likert scale, scored 0-4
    - Measure the difference in the average between the two groups at 2-months
- Breastfeeding ever at 4-months
    - Single item, yes or no
    - Measure the difference in caregivers reporting ‘yes’ at 2-months and 4-months
- Breastfeeding duration at 4-months
    - Single item, free text answer
    - Measure the difference in reported time (measured in months) spent breastfeeding each infant at 4-months
- Infant birthweight
    - Extracted from the EHR at time of NICU discharge
- Infant comorbidities
    - Extracted from the EHR at time of NICU discharge
    - Record comorbidities for each infant
- Infant NICU length of stay
    - Extracted from the EHR at time of NICU discharge
    - Measure the difference in infant NICU length of stay between the two groups, stratifying by gestational age category
- Infant reliance on durable medical equipment at discharge
    - Extracted from the EHR at time of NICU discharge
    - Record reliance on durable medical equipment, including home oxygen, feeding tube, ventilator, tracheostomy, etc.
- Infant number of readmissions and ED visits to the Children’s Hospital of Philadelphia within 3-months
    - Extracted from the EHR at 3-months after NICU discharge
    - Measure the difference in the composite outcome of inpatient readmissions and ED visits, inpatient readmissions alone, and ED visits alone between the two groups

V. **CONSORT Checklist: Information to include when reporting a pilot trial**

Section/topic and item No	Standard checklist item	Extension for pilot trials	Page No where item is reported
**Title and abstract**			
1a	Identification as a randomised trial in the title	Identification as a pilot or feasibility randomised trial in the title	Title
1b	Structured summary of trial design, methods, results, and conclusions (for specific guidance see CONSORT for abstracts)	Structured summary of pilot trial design, methods, results, and conclusions (for specific guidance see CONSORT abstract extension for pilot trials)	Manuscript structured in this format
**Introduction**			
Background and objectives:			
2a	Scientific background and explanation of rationale	Scientific background and explanation of rationale for future definitive trial, and reasons for randomised pilot trial	Introduction
2b	Specific objectives or hypotheses	Specific objectives or research questions for pilot trial	Introduction
**Methods**			
Trial design:			
3a	Description of trial design (such as parallel, factorial) including allocation ratio	Description of pilot trial design (such as parallel, factorial) including allocation ratio	Methods
3b	Important changes to methods after trial commencement (such as eligibility criteria), with reasons	Important changes to methods after pilot trial commencement (such as eligibility criteria), with reasons	Methods
Participants:			
4a	Eligibility criteria for participants		Methods
4b	Settings and locations where the data were collected		Methods
4c		How participants were identified and consented	Methods
Interventions:			
5	The interventions for each group with sufficient details to allow replication, including how and when they were actually administered		Methods and Supplement
Outcomes:			
6a	Completely defined prespecified primary and secondary outcome measures, including how and when they	Completely defined prespecified assessments or measurements to address each pilot trial objective specified in 2b, including how and when they were assessed	Methods and Supplement

	were assessed		
6b	Any changes to trial outcomes after the trial commenced, with reasons	Any changes to pilot trial assessments or measurements after the pilot trial commenced, with reasons	Methods
6c		If applicable, prespecified criteria used to judge whether, or how, to proceed with future definitive trial	N/A
Sample size:			
7a	How sample size was determined	Rationale for numbers in the pilot trial	Methods
7b	When applicable, explanation of any interim analyses and stopping guidelines		N/A
Randomization:			
Sequence generation:			
8a	Method used to generate the random allocation sequence		Methods
8b	Type of randomisation; details of any restriction (such as blocking and block size)	Type of randomisation(s); details of any restriction (such as blocking and block size)	Methods
Allocation concealment mechanism:			Methods
9	Mechanism used to implement the random allocation sequence (such as sequentially numbered containers), describing any steps taken to conceal the sequence until interventions were assigned		Methods
Implementation:			
10	Who generated the random allocation sequence, enrolled participants, and assigned participants to interventions		Methods
Blinding:			
11a	If done, who was blinded after assignment to interventions (eg, participants, care providers, those assessing outcomes) and how		Methods
11b	If relevant, description of the similarity of		Methods

	interventions		
Analytical methods:			
12a	Statistical methods used to compare groups for primary and secondary outcomes	Methods used to address each pilot trial objective whether qualitative or quantitative	Methods
12b	Methods for additional analyses, such as subgroup analyses and adjusted analyses	Not applicable	N/A
**Results**			
Participant flow (a diagram is strongly recommended):			
13a	For each group, the numbers of participants who were randomly assigned, received intended treatment, and were analysed for the primary outcome	For each group, the numbers of participants who were approached and/or assessed for eligibility, randomly assigned, received intended treatment, and were assessed for each objective	Figure 1
13b	For each group, losses and exclusions after randomisation, together with reasons		Figure 1
Recruitment:			
14a	Dates defining the periods of recruitment and follow-up		Methods
14b	Why the trial ended or was stopped	Why the pilot trial ended or was stopped	Methods
Baseline data:			
15	A table showing baseline demographic and clinical characteristics for each group		Table 1
Numbers analyzed:			
16	For each group, number of participants (denominator) included in each analysis and whether the analysis was by original assigned groups	For each objective, number of participants (denominator) included in each analysis. If relevant, these numbers should be by randomized group)	Table 1
Outcomes and estimation:			
17a	For each primary and secondary outcome, results for each group, and the estimated effect size and its precision (such as 95% confidence interval)	For each objective, results including expressions of uncertainty (such as 95% confidence interval) for any estimates. If relevant, these results should be by randomized group	Results
17b	For binary outcomes, presentation of both	Not applicable	N/A

	absolute and relative effect sizes is recommended		
Ancillary analyses:			
18	Results of any other analyses performed, including subgroup analyses and adjusted analyses, distinguishing prespecified from exploratory	Results of any other analyses performed that could be used to inform the future definitive trial	N/A
Harms:			
19	All important harms or unintended effects in each group (for specific guidance see CONSORT for harms)		None
19a		If relevant, other important unintended consequences	None
**Discussion**			
Limitations:			
20	Trial limitations, addressing sources of potential bias, imprecision, and, if relevant, multiplicity of analyses	Pilot trial limitations, addressing sources of potential bias and remaining uncertainty about feasibility	Discussion
Generalizability:			
21	Generalizability (external validity, applicability) of the trial findings	Generalizability (applicability) of pilot trial methods and findings to future definitive trial and other studies	Discussion
Interpretation:			
22	Interpretation consistent with results, balancing benefits and harms, and considering other relevant evidence	Interpretation consistent with pilot trial objectives and findings, balancing potential benefits and harms, and considering other relevant evidence	Discussion
22a		Implications for progression from pilot to future definitive trial, including any proposed amendments	Discussion
**Other information**			
Registration:			
23	Registration number and name of trial registry	Registration number for pilot trial and name of trial registry	Methods
Protocol:			
24	Where the full trial protocol can be accessed, if available	Where the pilot trial protocol can be accessed, if available	Supplement and ClinicalTrials.gov ID NCT05930327
Funding:			

25	Sources of funding and other support (such as supply of drugs), role of funders		Funding statement
26		Ethical approval or approval by research review committee, confirmed with reference number	Methods

VI. References

1. Eldridge SM, Chan CL, Campbell MJ, et al. CONSORT 2010 statement: Extension to randomised pilot and feasibility trials. *The BMJ*. 2016;355. doi:10.1136/bmj.i5239
